# Combination Therapy With Charged Particles and Molecular Targeting: A Promising Avenue to Overcome Radioresistance

**DOI:** 10.3389/fonc.2020.00128

**Published:** 2020-02-14

**Authors:** Katrien Konings, Charlot Vandevoorde, Bjorn Baselet, Sarah Baatout, Marjan Moreels

**Affiliations:** ^1^Radiobiology Unit, Belgian Nuclear Research Center (SCK•CEN), Mol, Belgium; ^2^Radiobiology, Radiation Biophysics Division, Department of Nuclear Medicine, iThemba LABS, Cape Town, South Africa; ^3^Department of Molecular Biotechnology, Ghent University, Ghent, Belgium

**Keywords:** radioresistance, radiosensitization, X-rays, proton therapy, particle therapy, carbon ion therapy, molecular targeted drugs, combination treatment

## Abstract

Radiotherapy plays a central role in the treatment of cancer patients. Over the past decades, remarkable technological progress has been made in the field of conventional radiotherapy. In addition, the use of charged particles (e.g., protons and carbon ions) makes it possible to further improve dose deposition to the tumor, while sparing the surrounding healthy tissues. Despite these improvements, radioresistance and tumor recurrence are still observed. Although the mechanisms underlying resistance to conventional radiotherapy are well-studied, scientific evidence on the impact of charged particle therapy on cancer cell radioresistance is restricted. The purpose of this review is to discuss the potential role that charged particles could play to overcome radioresistance. This review will focus on hypoxia, cancer stem cells, and specific signaling pathways of EGFR, NFκB, and Hedgehog as well as DNA damage signaling involving PARP, as mechanisms of radioresistance for which pharmacological targets have been identified. Finally, new lines of future research will be proposed, with a focus on novel molecular inhibitors that could be used in combination with charged particle therapy as a novel treatment option for radioresistant tumors.

## Introduction

Currently the main treatment options for cancer patients include surgery, chemotherapy, radiotherapy and immunotherapy. About 50% of cancer patients receive radiotherapy during the course of their treatment with the majority of patients being treated with conventional radiotherapy using photons (X-rays) (1). Since the start of treatment with photons, many technological advances and new treatment strategies have been implemented to optimize treatment delivery and to decrease the occurrence of side effects in healthy tissues. Unfortunately, despite these advances, resistance to radiotherapy, and recurrence of the disease is still observed.

Radioresistance of cancer cells implicates that to eradicate these cells, higher irradiation doses than the usual doses are needed. In theory, every tumor can be controlled if a sufficient high dose can be delivered, but in clinical practice, the radiation dose is unfortunately limited by the tolerance of the surrounding normal tissue (2). Resistance of cancer cells can be either “intrinsic” or “acquired.” Intrinsic resistance is naturally present within the cancer cell even before the treatment has started. Acquired resistance is induced by the irradiation itself and is a process in which the tumor cells or tissues adapt to the radiotherapy induced changes and develop radiation resistance (3, 4). Resistance to conventional radiotherapy is one of the major factors leading to radiotherapy failure, leading to cancer recurrences, metastases and a poor prognosis in cancer patients (5). Over the years, tremendous progress has been made to understand the treatment response of cancer cells and to improve the curative rate by specifically targeting of the DNA damage response pathways in order to obtain selective radiosensitization of cancer cells (6). However, despite the various efforts to overcome radioresistance, the concordant molecular mechanisms of cellular resistance to radiotherapy are still not completely understood.

**Figure 1:**
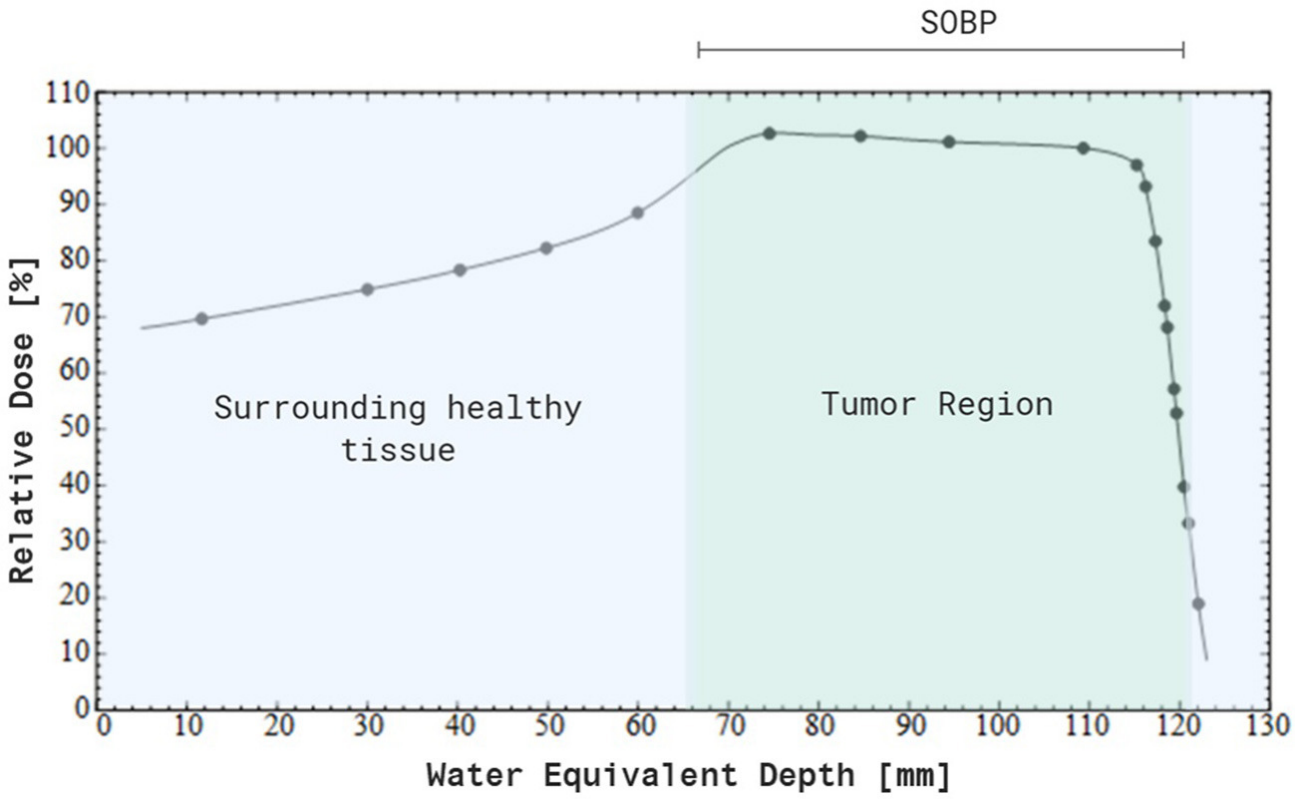
Percentage depth-dose distribution of a modulated 200 MeV proton beam, resulting in a spread-out Bragg peak (SOBP). Note that a maximum dose is delivered to the tumor tissue, while there is no dose deposited beyond the SOPB. In addition, a smaller dose is delivered to the entrance healthy tissue compared to the SOBP. Created with BioRender.

**Figure 2:**
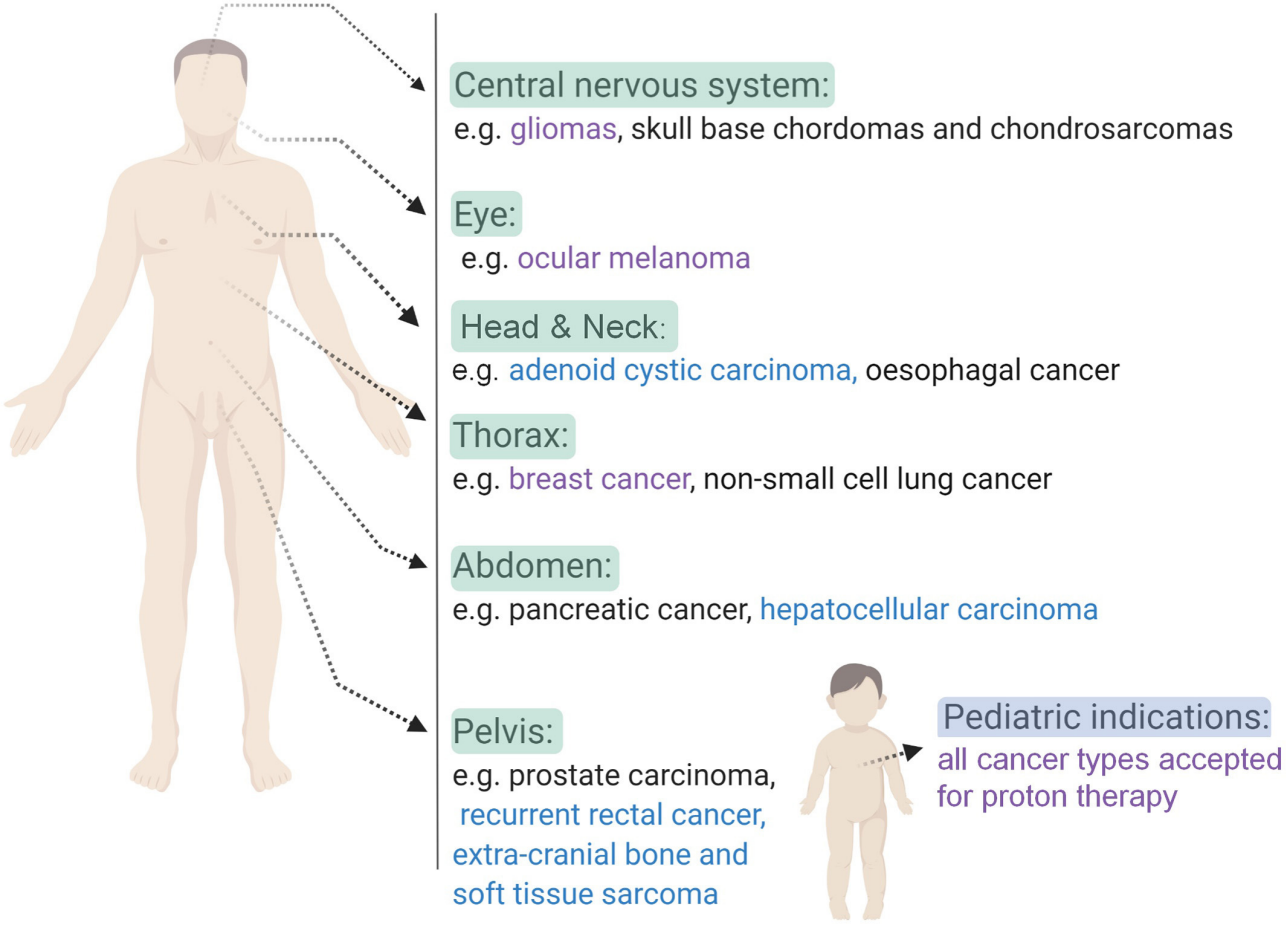
An overview of some of the clinical indications that can be treated with charged particle therapy based on ongoing clinical trials. Indications for proton therapy (purple), carbon ion therapy (blue) and both (black) charged particle therapies are listed per body side (16–18). Created with BioRender.

Nowadays, particle therapy is an emerging treatment modality in external beam radiotherapy, which makes use of charged particles such as protons or carbon ions to treat cancer (7, 8). Particle therapy has several advantages compared to conventional radiotherapy with photons, of which the most important one is the optimal dose distribution (9). Charged particles are characterized by a low entrance dose, followed by a high energy deposition near the end of their track in the so-called Bragg peak. This energy deposition can be specifically focused on the tumor, while almost no dose is delivered to the normal tissue beyond the peak (see Figure 1 for proton therapy). As a consequence, the healthy tissues surrounding the tumor will receive a lower irradiation dose compared to treatment with photons and it allows the treatment of lesions close to critical structures. This dosimetric advantage of charged particles could also allow the delivery of higher radiation doses to the tumor tissue, which might help to overcome radioresistance in specific tumor types. Another additional advantage of charged particles, specifically of carbon ions, is the increased relative biological effectiveness (RBE) of a factor 2–5 (9–11). The RBE is defined as the ratio of a photon (usually 250 kVp, >1 MeV X-rays, or Co-60 γ-rays) to charged particle dose that produces the same biological effect. The underlying reason for the increased RBE of carbon ions compared to photons is due to their higher linear energy transfer (LET). This means that carbon ion beams transfer their energy differently on a microscopic scale which results in a higher ionization density along the radiation track as compared to low-LET radiation types, such as photons and protons. A fixed RBE of 1.1 is currently adopted in clinical practice for protons, which is very similar to that of sparsely ionizing high-energy photons. However, considerable *in vitro* and *in vivo* studies indicate that the RBE of protons is significantly higher in the distal fall-off region of the Bragg peak, which gives rise to an ongoing debate on the implementation of a variable RBE in proton treatment planning (12). Table 1 gives an overview of the general RBE values which are applied in clinical practice for external beam radiotherapy, specifically for the radiation qualities relevant to this review. Although the majority of patients is still treated with conventional radiotherapy, the proportion of patients being treated by particle therapy is vastly increasing (14). Unfortunately, there still remains a lack of clinical prospective data to illustrate the benefit of charged particle therapy compared to conventional radiotherapy in order to fulfill evidence-based medicine requirements. Together with the high cost-effectiveness, this feeds some of the criticisms toward particle therapy. Despite these challenges, the clinical results of particle therapy are convincing and several new centers are under construction around the world. The patients statistics, published by the Particle Therapy Co-Operative Group in 2016, show that ~180,000 patients have been treated with particle therapy worldwide, with around 85% (± 150,000) of the patients being treated with protons and around 12% (± 22,000) with carbon ions (15). While carbon ion therapy is traditionally used for deep-seated hypoxic tumors that are adjacent to radiosensitive structures and is still considered to be an “experimental treatment,” this approach is slowly changing toward new clinical indications where the distinct signal response pathways of carbon ions is further exploited (16). An extensive number of randomized clinical trials on larger patient groups is currently ongoing for both charged particle therapy modalities, so the number of accepted indications for charged particle therapy will probably become more clear in the coming years. Based on a recent questionnaire of the European Organization for the Research and Treatment of Cancer (EORTC), the indications for treatment with charged particles in European particle therapy centers include soft tissues sarcomas, chordomas/chondrosarcomas, meningiomas, brain tumors (non-meningioma), head and neck tumors, and prostate tumors (some of these clinical indications are illustrated in Figure 2). Moreover, breast, lung and liver cancers can also be treated with particle therapy, however, this is only done in a minority of centers (13, 17).

**Table 1:** Overview of commonly reported relative biological effectiveness (RBE) values for radiation qualities that are used in external beam radiotherapy and within the scope of this review.

**Radiation quality**	**RBE**
Carbon-ions	2–5
Protons	1.1
Photons	1

*Note that these RBE values are currently applied in clinical practice, but they are under discussion and further experimentation and larger datasets are required to obtain more accurate RBE values (13)*.

As mentioned above, carbon ions have a higher RBE and thus greater cell killing effectiveness (19, 20). This can overcome radioresistance of hypoxic tumor regions, reduce the effect of fractionation and decrease cell cycle dependence of the radiation response (21). Hence it is expected that carbon ions can directly overcome radioresistance whereas this is less evident for protons. However, despite the fact that photons and protons are both low-LET radiation types and are considered to have a similar RBE, an increasing amount of studies show that protons actually produce different biological effects compared to photon irradiation as reviewed by Girdhani et al. (22, 23). Although much research has been performed concerning the physical aspects of both protons and carbon ions, the uncertainties concerning the biological aspects of particle irradiation calls for further investigation. One challenging question is whether the physical differences between photons and charged particles are also reflected in a differential biological response, which might also affect the underlying mechanisms of resistance to radiotherapy.

It is already known from previous studies that many different factors are associated with radioresistance of cancer cells and multiple reviews have already described some of the possible mechanisms underlying radioresistance during conventional radiotherapy (24, 25). Examples are cancer stem cells (CSCs) and hypoxia, as well as perturbations in survival pathways, DNA damage repair pathways, developmental pathways (24, 25). In contrast, the knowledge about the impact of particle therapy on radioresistance is scarce. Therefore, this targeted review will give an overview of selected literature related to the impact of charged particle irradiation on therapeutic radioresistance. We will specifically focus on the role of hypoxic regions and CSCs as well as some molecular pathways involved in radioresistance for which pharmacological targets have been identified. In addition, new lines of future research will be proposed with a special emphasis on novel molecular inhibitors and treatment strategies where charged particle therapy could be beneficial over conventional radiotherapy. This overview aims to illustrate the great potential of particle therapy in the treatment of radioresistant tumors.

## Selected Mechanisms of Radioresistance

### Hypoxia

Tumor hypoxia is one of the well-described factors that can lead to resistance to conventional radiotherapy. Because photons induce most of their damage indirectly by the formation of free radicals, the oxygen level in the tumor plays a crucial role in the success of radiotherapy, which is also known as the oxygen effect. Hence, the lower levels of oxygen in hypoxic tumor regions will decrease the damaging effect of photon radiation on cells (26). Several studies have already reported the link between hypoxia and radioresistance, both *in vitro* and *in vivo* (27–29).

**Figure 3:**
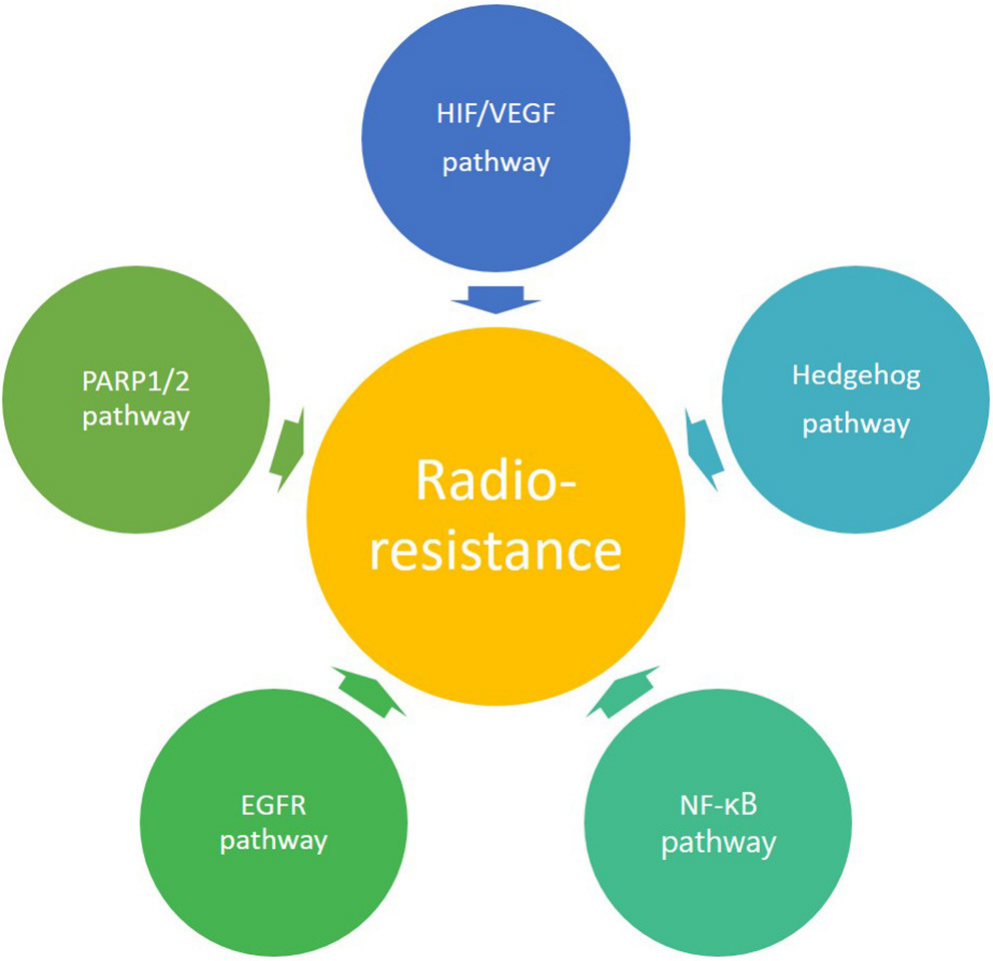
Overview of the molecular signaling pathways involved in radioresistance.

Besides the influence of oxygen on the induction of DNA damage by photon irradiation, tumor hypoxia itself also affects different molecular pathways. An important regulator in the response to hypoxia is hypoxia inducible factor 1 (HIF-1), which plays a key role in the radioresistance of hypoxic tumors (Figure 3) (30–33). HIF-1 is a heterodimer consisting of two subunits, an α-subunit (HIF-1α) and a β-subunit (HIF-1β). The expression of HIF-1α is dependent on oxygen levels, and is induced under hypoxic conditions, whereas HIF-1β is constitutively expressed (24). In this context, it has been observed that photon radiation induces HIF-1 expression in solid tumors (34, 35). Upon activation, HIF-1 can increase the expression of different target genes involved in growth, energy metabolism, endothelial cell function and neovascularization, thereby promoting tumor growth (36, 37). These hypoxia-induced changes could eventually give rise to metastasis of cancer cells (38, 39). In conclusion, hypoxia is known to be a negative prognostic and predictive factor, and it is generally acknowledged as a major limitation for tumor control in conventional radiotherapy resulting in a poor clinical outcome (40).

In contrast, the effect of high-LET ionizing radiation on cancer cells, such as carbon ions, depends much less on the presence of oxygen (26). This is due to the fact that high-LET radiation primarily induces complex damage directly to the DNA, which is more difficult to repair. As a consequence, high-LET radiation can eradicate hypoxic tumor cells more effectively than low-LET radiation (41–44). This is also reflected in the oxygen enhancement ratio (OER), defined as the ratio of the dose needed in hypoxic cells divided by the dose needed in normoxic cells to obtain the same biological effect. The OER decreases with increasing LET, for example, the OER for low-LET radiation such as photons and protons is around 2.5–3.0, whereas the OER for carbon ions is around 1.6–2.0 (42, 45). Since the OER of protons is similar to photons, the advantage of proton therapy to overcome hypoxia-induced radioresistance is less obvious than for carbon ions. Both *in vitro* and *in vivo* evidence demonstrates that carbon ion irradiation is able to reduce hypoxia-induced radioresistance (46–48). Moreover, ions heavier than carbon, such as nitrogen and oxygen ions, could have additional advantages, specifically for hypoxia-induced radioresistant tumors (49).

The effect of charged particle irradiation on the molecular pathways affected by tumor hypoxia, specifically HIF1-α expression, is underexplored. Despite the limited number of available studies, the first results illustrate that proton irradiation is able to decrease HIF-1α and VEGF expression *in vitro* in different cell types compared to non-irradiated controls (50). This was in sharp contrast to photon irradiation, where VEGF and HIF-1α were upregulated in a dose-dependent manner. Another *in vitro* study illustrated that proton irradiation was able to induce similar levels of apoptotic cell death in both hypoxic and normoxic cells, but only for two of the three investigated cell lines (51). The same effect could not be observed for photons. An *in vivo* study using an orthotropic breast cancer model reported lower levels of VEGF in breast tumors irradiated with protons compared to non-irradiated controls (52). However, this effect was only observed at a high proton dose of 30 Gy and not compared to the effect of similar photon doses (52). Carbon ion irradiation is also able to decrease the expression of the HIF-1α subunit in hypoxic conditions, both *in vitro* and *in vivo* (53, 54). For VEGF on the other hand, conflicting results exist about the response to carbon ion irradiation. Some studies observed no altered VEGF expression while others observed an increased VEGF expression after carbon ion irradiation (55–57). Until now, clinical evidence of increased hypoxic tumor control with proton therapy is still lacking and only one study with high-LET carbon ions has been published so far. In this study of Nakano et al. patients with stage III and IV cervical tumors were treated with carbon ion irradiation. After treatment, patient follow-up showed a similar disease-free survival and local control in both patients with hypoxic and normoxic tumors (41). Overall, current data for proton and carbon ion irradiation suggest that both radiation types can counteract the hypoxia-induced radioresistance more efficiently compared to conventional photon irradiation.

### Cancer Stem Cells

**Figure 4:**
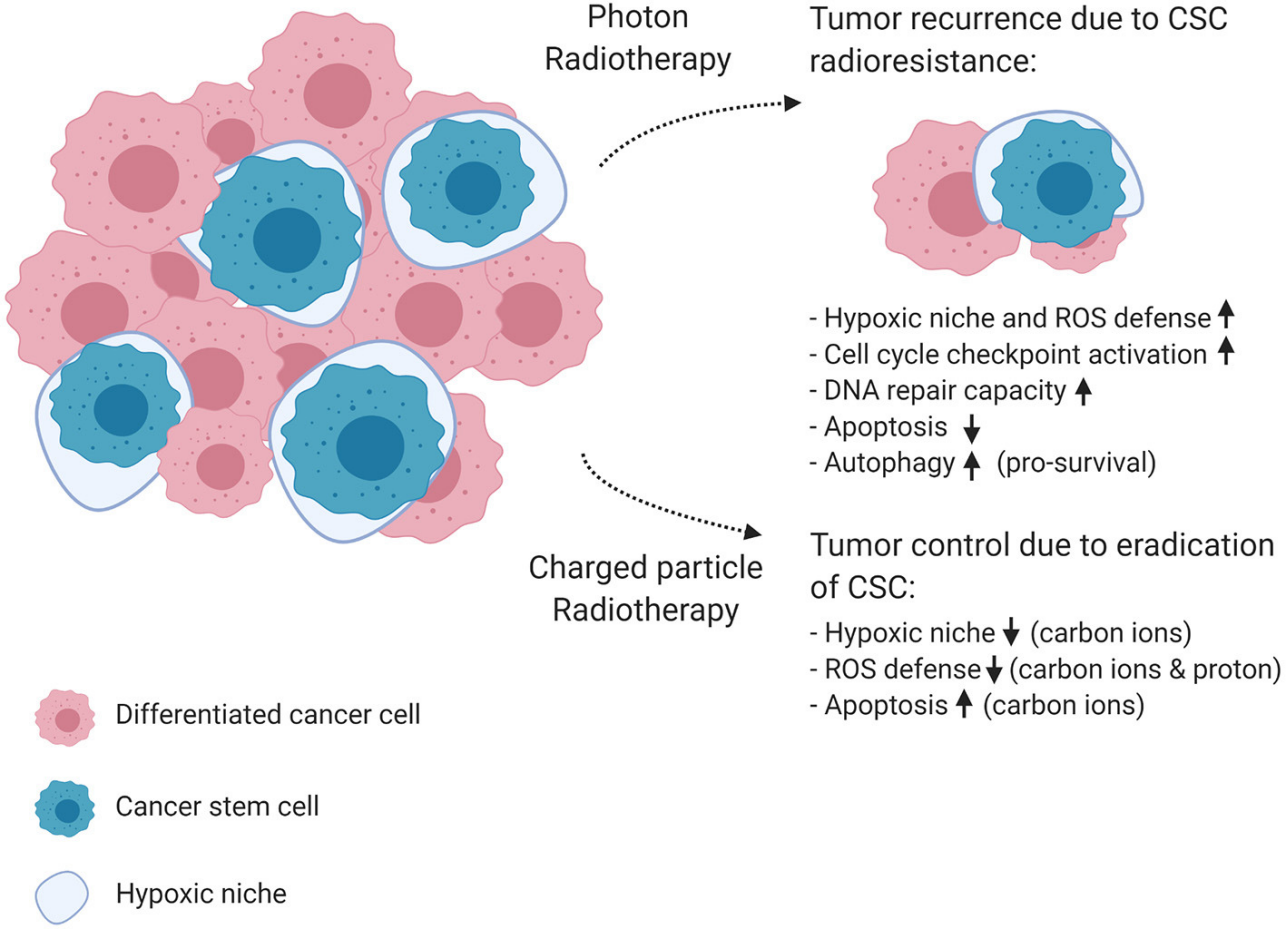
The effect of photon and charged particle irradiation on cancer stem cell radioresistance. This schematic diagram shows the mechanisms of cancer stem cell resistance to conventional photon radiotherapy on the top right corner of the figure, resulting in tumor recurrence and metastasis. In the bottom right corner, the diagram shows how charged particle therapy could improve tumor control. Carbon ions were able to depress pro-survival signaling, which results in enhanced apoptosis, and are known to have a lower OER, which makes them more effective against the protective hypoxic niche of CSCs. In addition, protons generated higher ROS levels in CSCs, resulting in increased cell killing compared to photons. Created with BioRender.

CSCs, also referred to as cancer initiating cells, were first identified in 1997 by Bonnet and Dick in acute myeloid leukemia and later in many solid tumors (58). CSCs are defined as a small subset of cancer cells, which constitute a reservoir of self-sustaining cells with the unlimited potential to self-renew and maintain the tumor (59). In addition, CSCs display an innate resistance to chemotherapeutic agents and conventional radiotherapy, and are therefore believed to play an important role in treatment failure and recurrence. This assumption directly implies that an anti-cancer therapy can only cure a tumor if all CSCs are killed (60). In addition, several studies observed an increase in the absolute number of CSCs in the tumor bulk after conventional radiotherapy (61). The mechanisms of radioresistance in CSCs compared to non-CSCs were firstly described for glioblastoma multiforme and breast cancer. Here, the observed radioresistance of CSCs appeared to be related to differences in DNA-repair capacity due to constitutive phosphorylation of the DNA checkpoint kinases Chk1 and Chk2 (62), their quiescent state and the hypoxic niche in which CSCs reside together with the enhanced ROS defenses in response to conventional radiotherapy (63, 64). Some studies failed to illustrate the difference in DNA repair capacity between glioma cells and glioma CSCs (65), while others could demonstrate an enhanced ataxia-telangiectasia mutated-dependent DNA DSB repair proficiency (66, 67). Next to these mechanisms, other studies illustrated that the activation of survival signaling pathways, such as anti-apoptotic Bcl-2 and PI3K/Akt/mTOR, contribute to the radioresistance of CSCs (68, 69). Figure 4 highlights the hallmarks of radioresistance in CSCs, including intrinsic determinants such as their increased DSB repair capacity, enhanced cell-cycle checkpoint activation, reduced apoptosis and increased autophagy as a pro-survival mechanism to maintain homeostasis. As previously mentioned, CSCs are preferentially localized in hypoxic niches of the tumor microenvironment (70). Therefore, the hypoxic niche is considered to be an important extrinsic determinant that can potentially maintain or enhance the stem cell phenotype of cancer cells and contribute to the emergence of metastatic clones (71).

So far, the evidence regarding the applicability of charged particle therapy to overcome radioresistance of CSCs is growing. A recent *in vitro* study has shown that proton irradiation preferentially targets CSCs and increases ROS levels in treatment of resistant non-small cell lung cancer (NSCLC) to a greater extent than photons of the same dose (72). Similar results were obtained in a study using patient-derived glioma stem cells, where proton irradiation generated greater cytotoxicity in glioma CSCs compared to photon radiation resulting in reduced clonogenic survival fractions after proton irradiation (73). The underlying mechanism by which proton radiation eradicates glioma stem cells more efficiently, might be the increased production of ROS that induces greater DNA damage, cell cycle alterations and cell apoptosis in glioma stem cells. In this study, the authors could demonstrate that photon radiation produces smaller quantities of ROS in CSCs compared to proton radiation, while a ROS scavenger could abolish proton radiation-induced ROS generation. In another study, proton radiation appeared to be more efficient to kill breast CSCs compared to the same dose of photons, resulting in lower cell survival and higher DNA damage (74). Both studies support the enhanced ability of proton irradiation to overcome CSC radioresistance compared to photon irradiation.

In theory, the higher RBE of carbon ions and their ability to induce DNA damage mostly through direct interaction regardless of oxygen levels, should make them more effective against the hypoxic niche and enhanced ROS defense of CSCs (75). A first study could show the advantage of carbon ion therapy over conventional radiotherapy for putative colon cancer stem-like cells (76). In a more recent study, carbon ions appeared to reduce the number of colonies and spheroids in pancreatic cancer stem-like cells compared to photon irradiation (77). This is in line with previously published results that also illustrated a superior capacity to kill pancreatic cancer stem-like cells by carbon ions, which corresponded to an increased number of residual γ-H2AX foci compared to photons (78). Furthermore, an enhanced OER and cell killing could be observed after carbon ion irradiation compared to photons in resistant head and neck squamous cell carcinoma (HNSCC) CSCs (53). Recently, the same authors postulated that the distribution of ROS produced after photon irradiation can have an influence on leading to invasion/migration-related mechanisms: a uniform distribution of ROS after photon irradiation was able to induce mechanisms leading to invasion/migration, while concentrated ROS during carbon ion irradiation was unable to trigger invasion/migration mechanisms (79). No difference between hypoxic and normoxic conditions on cell killing could be observed after carbon ion irradiation, while all cell lines were more resistant to photon irradiation under hypoxic conditions. These observations support the greater efficiency of carbon ions to overcome radioresistance of CSCs residing in hypoxic niches. Other studies could already demonstrate a better efficacy of heavy ions to overcome pro-survival signaling (such as AKT survival signaling), suggesting that carbon ions could enhance apoptosis in radioresistant CSCs (80). And the evidence keeps on growing with a recent study showing the efficiency of carbon ions to eradicate radioresistant patient-derived glioma CSCs, leading to growth inhibition and prolonged survival in mice (81). However, there was one contradictory study showing an extended cell cycle arrest in response to both photon and carbon ion irradiation, indicating that carbon ion irradiation was not able to depress cell cycle checkpoint activation in CSCs, making them resistant to both photons and carbon ions (82).

### Signaling Pathways Involved in Radiation Resistance

Resistance to photon radiation can, apart from hypoxia and the presence of CSCs, also be influenced by specific signaling pathways. In contrast to photon radiotherapy, the modulation of these signaling pathways in response to charged particle radiation has only been studied to a limited extent. In the following part, a comparison is made between the effect of photon and charged particle irradiation on the signaling pathways that are known to be involved in resistance of tumor cells to conventional radiotherapy (Figure 3, Table 2). While there are a large number of signaling pathways that are involved in radioresistance, this review will particularly focus on those pathways for which pharmacological targets have been identified, which led to the development of small molecule inhibitors which will be discussed in part 3. This could provide a roadmap to uncover potential mechanisms and therapeutic indications where charged particle therapy could be used instead of conventional radiotherapy in order to overcome radioresistance.

**Table 2:** Overview of the different experimental parameters used in the studies with particle-irradiated cancer cells.

**Type of irradiation**	**Energy**	**LET (keV/μm)**	**Dose (Gy)**	**Dose rate**	**Experimental model**	**References**
**Hypoxia/HIF/VEGF**						
Carbon ion	290 MeV/u	Ranged from 14 to 74 depending on depth	16	3 Gy/min	NFSa fibrosarcoma cells implanted in hind leg of mice	(43)
Carbon ion	NA	NA	~1, 2, 4, or 6	NA	A549, NCI-H1437 (Human lung cancer cells)	(44)
Carbon ion	290 MeV/u	18, 43, 50, and 74	0–10	0.037 and 1 Gy/min	SCC VII tumors in hind leg of mice (Murine squamous cell carcinoma cells)	(47)
Carbon ion	140–170 MeV/u	Mean dose average LET:75 (64-96)	33 and 37	NA	R3327-H, -HI, and -AT1 (Syngeneic Dunning prostate adenomacarcinomas) implanted in thigh of rats	(48)
Carbon ion	NA	100 and 150	0–6	NA	CHO-K1 and RAT-1 (Dunning rat prostate cancer cells)	(49)
Proton	1 GeV/nucleon	≈0,24	0.5, 1, and 2	0.25–0.33 Gy/min	A549 (Human lung cancer cells) and HMVEC (Human Lung Microvascular Endothelial Cells)	(50)
Proton	35 MeV	NA	10	2.31 Gy/sec	LLC, Molt-4 human leukemia cells and HepG2 human hepatocelllar carcinoma cells	(51)
Proton	100 MeV	NA	Cells: 2, 4, 8, and 16 Mice tumors: 10, 20, and 30	NA	4T1 murine breast cancer cells + implanted in mice	(52)
Carbon ion	72 MeV/n	33, 6	10	2 Gy/min	SQ20B, SQ20B-CSCs, and FaDu (Human squamous cell carcinoma)	(53)
Carbon ion	120, 45–135, 16 MeV/u (cells) 122,36–183,74 MeV/u	mean dose average of LET 50–70	2	NA	A549 and H1299 (Human lung cancer cells)	(54)
Carbon ion	290 MeV/u	13.3, 50, and 90	15	~7.2 Gy/min	RERF-LC-AI (Squamous cell lung carcinoma)	(55)
Carbon ion	9.8 MeV/u (on target)	170	2	NA	A549 (Human lung cancer cells) and HUVECs (Human umbilical vein endothelial cells)	(56)
Carbon ion	350 MeV/u	15,4	2, 4, and 8	0.5 Gy/min	C6 (Human glioma cells) and HMEC-1 cells (Human microvascular endothelial cells)	(57)
Proton	62 MeV	NA	12 and 16	15 Gy/min	HTB140 (Human melanoma cells)	(83)
Carbon ion	75 MeV/n	33.6	1, 2, 3, 4, and 5	NA	SQ20B (Human squamous cell carcinoma)	(84)
**Cancer stem cells**						
Proton	Therapeutic proton beam	NA	2, 4, and 8	NA	CSC-enriched cells from therapy-resistant human H460 and A549 (Human lung cancer cells)	(72)
Proton	Therapeutic proton beam	NA	5 and 10	2	IN528 and T4213 (Patient-derived glioma stem cells)	(73)
Proton	7.5 MeV	2	2 and 4	0.24 Gy/sec	CSC and non-CSC enriched from MCF-7 cells (Human breast cancer cells)	(74)
Carbon ion	290 MeV/n	50	Cells: 1, 2, 4, and 6 Tumors: 5, 15 and 30	NA	CSC and non-CSC enriched from HCT116 and SW480 (Human colon cancer cells)	(76)
Carbon ion	290 MeV/n	50	1–6	NA	CSCs and non-CSCs isolated from PK45, PNAC1, MIAPaCa-2, and BxPc-3 (Human pancreatic cancer cells)	(77)
Carbon ion	290 MeV/n	13	2 and 10	2 Gy/min	SQ20B, SQ20B-CSC's, and FaDu (Human squamous cell carcinoma)	(79)
Carbon ion	290 MeV/n	50	1, 2, and 3	NA	CSC and non-CSC isolated from MIA PaCa-2 and BxPc-3 (Human pancreas cancer cells)	(78)
**EGFR pathway**						
Proton	35 MeV	NA	6	2.31 Gy/sec	H460, H1299 (Human lung cancer cells)	(85)
Proton	35 MeV	NA	0.5, 2, and 8	NA	MDA-MB-231 (Human breast cancer cells)	(86)
Proton	35 MeV	NA	0.5, 2, 8, and 16	2.31 Gy/sec	HT-29 (Human colon cancer cells)	(87)
Carbon ion	120–135 MeV/nucleon	dose-averaged LET ≈ 100	2 and 6	NA	wild-type EGFR, U87 EGFR++ and LN229 EGFR++ (Human glioblastoma cells)	(88)
Carbon ion	290 MeV/nucleon	50 keV/μm (middle of SOBP)	0.25, 1, and 5	NA	A549 (Human lung cancer cells)	(89)
Carbon ion	165 and 290 MeV/nucleon	Dose-averaged LET 13 or 75	2	NA	HeLa (Human cervical cancer cells)	(90)
Carbon ion	290 MeV/nucleon	50 (middle of SOBP)	2, 4, and 6	NA	A427, A549, H1299, H1650, H1703, H1975, H460, H520, H522, HCC827, LK2,II-18, H157, Ma-24, PC9, A549-WT, –ΔE746-A750, and –L858R (Human lung cancer cells)	(91)
Pulsed proton beam	45 MeV	NA	4, 8, 10, and 12	1 Gy/pulse	MCF-7, MDA-MB-231 (Human breast cancer cells)	(92)
Carbon ion	NA	122.36–136.92 MeV/u	0.125, 0.5, 1, 2, and 3	0.5 Gy/min	Hep3B, HepG2, PLC, and HuH7 (Human hepatic tumor cells)	(93)
**NF-κB pathway**						
Carbon ion	62 MeV/n	197 and 382 (at both positions used)	2, 4, 8, 12, and 16	11.45 ± 0.31 Gy/min	HTB140 (melanoma)	(94)
**PARP**						
Carbon ion	NA	13–100	2	~3 Gy/min	Ca9-22 (Human gingival squamous cell carcinoma)	(95)
Proton	160 MeV	4.3 (Bragg peak)	1, 2, 4, and 6	1 Gy/min	A549 (Human lung cancer cells) MIA PaCa-2 (Human pancreatic cancer cells)	(96)
Carbon ion	62 MeV [5.2 MeV/u]	entrance LET 290	1, 2, and 4	Flux: 2 × 10^5^ particles/cm^2^/sec	HeLa (Human cervix adenocarcinoma)	(97)
Carbon ion	290 MeV/nucleon	13 and 70	1, 3, and 5	1.2 Gy/min	MIA PaCa-2 (Human pancreatic cancer cells)	(98)
Carbon ion	NA	50	2	NA	R633 and TG1 (Human glioblastoma CSC)	(99)
Carbon ion	290 MeV/n	13 keV/μm		NA	CHO wild type and repair deficient mutants (Chinese hamster ovary cells)	(100)
Carbon ion	62 MeV [5.2 MeV/u]	entrance LET 287	1, 2, and 4	Flux: 2 × 10^5^ particles/cm^2^/sec	HeLa (Human cervix adenocarcinoma)	(101)
**Hh pathway**						
Carbon ion	95 MeV/n	73 KeV/μm	0, 0.25, 0.5, 1, 2, 3, and 4	NA	PC3 and DAOY Prostate cancer cells Pediatric medulloblastoma cells	(102)
Proton	200 MeV/n	3.96 ± 0.20 keV/μm	0.25, 0.5, 2, 4, and, 6	NA	PC3 and DAOY Prostate cancer cells Pediatric medulloblastoma cells	(102)

*LET, linear energy transfer; MeV, mega electron volt; HIF, hypoxia-inducible factor; VEGF, vascular endothelial growth factor; EGFR, epidermal growth factor receptor; PI3K, phosphoinositide 3-kinase; MAPK, mitogen-activated protein kinases; PARP, Poly (ADP-ribose) polymerase; CSC, cancer stem cells; NA, not available*.

#### EGFR Signaling Pathway

One of the most important pathways involved in cell survival, growth, proliferation and differentiation is the epidermal growth factor receptor (EGFR) pathway. The transmembrane protein EGFR is an important modulator in photon radioresistance. In this context, a positive correlation has been reported between the expression of EGFR and resistance to photon irradiation (103–105). Photon radiation can trigger the activation of EGFR, which in turn activates other downstream pathways that modulate cell processes, including cell migration, angiogenesis, apoptosis and invasion (Figure 3) (106–108). One of the downstream pathways is the phosphoinositide 3-kinase (PI3K)/AKT/mammalian target of rapamycin (mTOR) pathway, known to be one of the most commonly activated signaling pathways in cancer, leading to cell proliferation, survival and differentiation. Besides activation by EGFR, the PI3K/AKT/mTOR pathway can also become activated by photon radiation directly (109). Activation of EGFR induces the phosphorylation of PI3K which in turn activates AKT (110, 111). AKT is one of the key players in the PI3K pathway since it has many different downstream targets such as mTOR, NFκB, DNA-PK, Bad and many others. More specifically, activation of the PI3K/AKT/mTOR pathway results in the protection of tumor cells by decreased apoptosis and autophagy and increased activation of DNA repair molecules. In addition, the epithelial-mesenchymal transition (EMT) is stimulated. This cascade of events triggered by PI3K/AKT/mTOR pathway eventually leads to photon radioresistance (112–114). An active PI3K pathway has been implicated in the radioresistance of many different tumor types, for example acute myeloid leukemia (115), prostate (116–118), head and neck (119), brain (120) and lung (121, 122) cancers. Another pathway activated by EGFR is the RAS/RAF/MAPK pathway. Besides activation by EGFR and photon irradiation, the MAPK pathway can also become activated by mutations in the RAS proteins (121). Mutated RAS has been linked to the resistance of cancer cells to photon irradiation (123–127) which is caused by the downstream targets of RAS including the MAPK and the PI3K pathway. These targets are pro-proliferation and pro-survival, respectively, and an increased activity of both pathways has also been associated with radiation resistance (109, 114, 121, 123, 128).

In the context of particle radiation, different and contradicting responses of the EGFR pathway and its downstream targets have been observed. Data showed that proton irradiation is able to increase EGFR expression as well as to activate the MAPK pathway, in this way protons might induce radioresistance (51, 85, 129). In contrast, some studies have observed a decreased phosphorylation of AKT and MAPK together with an inhibited AKT signaling after proton exposure, which points to a decrease in radioresistance (86, 87). For carbon ion irradiation, Stahler et al. observed no activation of EGFR and downstream targets AKT and ERK1/2 (88). In contrast, photon radiation induced an increased activation, which supports again the fact that carbon ions are superior to photons. In addition, other studies observed decreased activation of the EGFR and PI3K/AKT/mTOR pathway after carbon ion irradiation (89, 90). Only the study of Ogata et al. included photon irradiation experiments and used iso-effective doses for carbon ions. This study could demonstrate that even low doses of carbon ions reduced the levels of phosphorylated AKT in human lung adenocarinoma cells, in contrast to photon irradiation where this effect was not observed. Interestingly, the EGFR mutational status can be used to predict the response to carbon ion irradiation. More specifically, a wild-type EGFR status was linked to a higher RBE compared to an EGFR-mutated status in a NSCLC cell line. Therefore, patients without EGFR mutations could benefit more from carbon ion irradiation (91). Overall these data suggest that carbon ion irradiation is more likely to inhibit the EGFR pathway and its downstream target pathways. The data on proton irradiation are less clear and requires further investigation.

#### NFκB-Pathway

The NFκB signaling pathway is activated by a number of different stimuli, including exposure to photon radiation, thereby playing an important role in radioresistance (130–132). Resistance to radiation occurs since the activation of the NFκB pathway results in the transcription of genes involved in evasion of apoptosis, proliferation, cell cycle, metastasis, invasion, and inflammation (24, 130, 133). Besides activation by photon irradiation, the NFκB pathway can be constitutively activated through mutations in the NFκB proteins, which play an important role in intrinsic radioresistance (133).

The knowledge about the impact of particle radiation on the NFκB-signaling pathway is limited. Proton beam irradiation was found to suppress the phosphorylation of NFκB in breast cancer cells, through the inhibited activation of AKT (86). In contrast, no changes were observed in NFκB expression in proton-irradiated liver cancer cells (134). However, another study found an *in vivo* activation of NFκB in mouse bone marrow cells after whole body proton irradiation, with a persistent activation of NFκB up to 1 month after irradiation (135). Similar effects were observed for the mice that were treated with whole body photon irradiation. In non-cancerous cells (HEK 293), Hellweg et al. observed that carbon ion irradiation was able to activate the NFκB pathway similar to photon irradiation (136, 137). In addition, activation of the NFκB pathway and its downstream target genes was found to be highest for heavy ions with an LET between ~50–200 keV/μm (138). Moreover, another study demonstrated significant activation of NFκB as early as 3 h after carbon ion irradiation, with a peak at 6 h after irradiation (139). Jelena et al. also found an increase in the protein levels of NFκB after carbon ion irradiation, while no changes were observed in the mRNA levels of NFκB in the same melanoma cells (94). No comparison was made with photon irradiation in these experiments. Taken together, the existing data indicates that carbon ion irradiation increases NFκB expression in a LET-dependent manner and thus could enhance radioresistance. In this regard, the NFκB pathway could therefore be a good target for inhibition in combination with carbon irradiation.

#### DNA Damage Signaling Involving Poly(ADP-Ribose) Polymerase (PARP)

Poly(ADP-ribose) polymerase or PARP is an important factor in DNA damage signaling due to its role in the repair of single strand breaks as well as DSBs. PARP is one of the first molecules to respond to DNA damage by binding to the DNA break, which eventually leads to the recruitment of other DNA repair molecules (140). In addition, cleaved PARP is an indicator of apoptosis. An increased expression of PARP has been observed in several tumor types and has been linked to drug resistance and increased survival of genotoxic stress (141–145) as well as the promotion and maintenance of cancer stemness (146–149). In some animal studies, PARP has also been linked to the survival response after photon radiation exposure, since the use of a PARP inhibitor in combination with photon radiation was able to enhance the effect of photon radiation, by decreasing cell growth and cell survival (150–153).

Although only a limited number of studies have investigated changes in the expression of PARP after particle irradiation, many have used PARP inhibitors in order to see if this approach could have sensitizing effects in combination with particle irradiation. The use of PARP inhibitors in combination with particle radiation will be explained in more detail in section inhibition of PARP. Other studies that investigated cleaved PARP levels after proton irradiation, observed an increased and more sustained PARP cleavage compared to photon radiation, both in normoxic as well as hypoxic environments (51, 73). In addition, carbon ions have been found to induce an LET dependent increase in cleaved PARP (95). Furthermore, PARP deficient cells were found to have the largest decrease in OER after carbon irradiation (100). From the current data it can be summarized that particle irradiation has a more pronounced effect on PARP expression and cleavage compared to photon irradiation. The inhibition of PARP could enhance the effect of particle radiation on PARP-mediated radioresistance even more, which makes it a good target for radiosensitization in future treatment strategies.

#### Hedgehog Pathway

The Hedgehog (Hh) signaling pathway is a differentiation pathway that is active during embryonal development as well as in adults during stem cell maintenance, tissue repair and regeneration (154–156). However, an aberrant signaling of this pathway has been implicated in the development and progression of several different tumor types (157–159). Activation of the Hh pathway induces the transcription of genes involved in cell cycle progression, apoptosis, angiogenesis and EMT. Moreover, it has become apparent that the Hh signaling pathway plays a key role in the regulation of CSC, such as their self-renewal capacity (160, 161).

An active Hh pathway has been linked to the resistance of cancer cells to photon radiation. More specifically, Chen et al. observed that in response to photon radiation the secretion of soluble sonic Hedgehog (SHH) was induced which lead to a radioprotective effect in hepatocellular cancer cells. In addition, the Hh pathway was found to become activated after photon exposure (162, 163). Furthermore, several other groups have reported a link between the Hh pathway and photon radioresistance in different cancer cell lines (164–167). Clinically, Sims-Mourtada et al. observed that activation of the Hh pathway could sustain esophageal tumor repopulation after chemo-radiation (164). Other clinical studies were able to show that Hh pathway activation was related to decreased disease-free survival, progression-free survival and overall survival (168, 169). To the best of our knowledge only two studies looked into the effect of particles on the Hh pathway. Recently, we showed that carbon ions were more effective in inducing significant alterations in the Hh pathway than photons (102) and that carbon ions in combination with a Hh inhibitor was more efficient in decreasing migration of MCF-7 breast cancer cells than X-ray irradiation (170).

## Potential Targets for Sensitizing Cancer Cells to Charged Particle Irradiation

From the overview given above, it is clear that there are many different players that can lead to radioresistance. Different signaling pathways are closely intertwined with hypoxia and CSCs, which contribute all together to radioresistance and eventually recurrence and metastasis. As illustrated above and in Table 2, a growing body of scientific evidence indicates that charged particles modulate the activity of some of these molecular pathways differently than photons. In addition, while CSCs have distinct signaling pathways that can regulate their radiation response differently compared to non-CSCs, proton and carbon ion irradiation seems promising to overcome CSC radioresistance due to the increased production of ROS and their impact on the hypoxic microenvironment, respectively.

Although the mechanisms of radioresistance are not fully uncovered, the described signaling pathways below present promising targets for inhibition in order to sensitize cancer cells to ionizing radiation. So far, several novel molecular agents have been developed to inhibit specific steps of these pathways involved in radioresistance to conventional radiotherapy. However, little is known on their use in combination with charged particle radiation. Besides targeting molecular pathways, adjustments can be made to the radiotherapy delivery by applying dose- or LET-painting, in order to overcome tumor hypoxia. In this way the physical and biological advantages of particle therapy can be exploited to the fullest and used to overcome therapy resistance.

**Figure 5:**
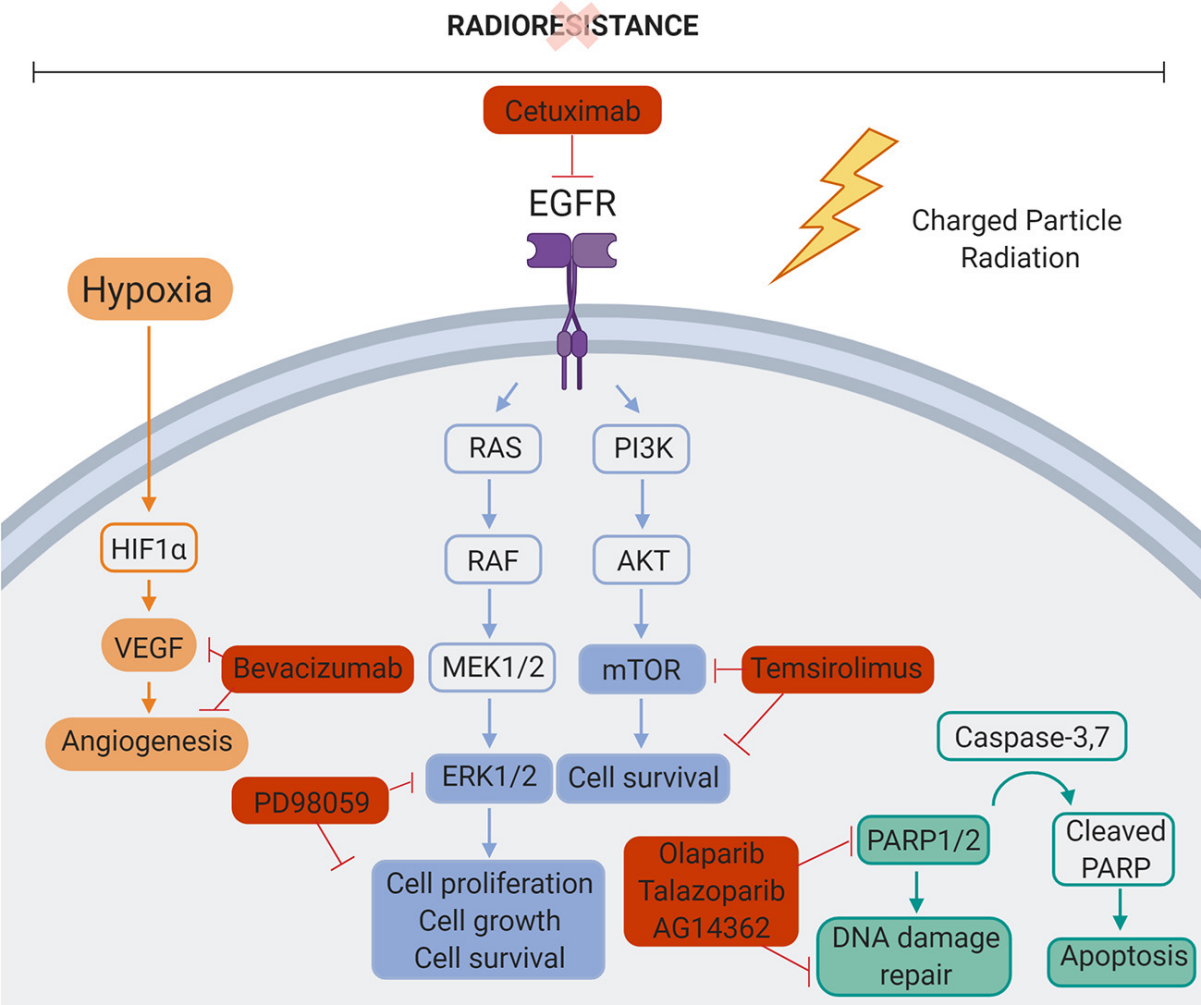
Molecular targets inhibited in combination with particle therapy. Mechanisms of cancer cell radiation resistance could be overcome by combining charged particle therapy and molecular targeting of the different signaling pathways involved in cancer cell radiation resistance. Bevacizumab, VEGF inhibitor. PD98059, ERK1/2 inhibitor. Cetuximab, EGFR inhibitor. Temsirolimus, mTOR inhibitor. Olaparib/Talazoparib/AG14361, PARP1/2 inhibitor. Created with BioRender.

In this part, we will focus on what is currently known on the use of pathway inhibitors in combination with charged particle therapy (Table 3). In addition, inhibitors that show promising results in conventional radiotherapy will be discussed for potential use with charged particle therapy (Figure 5). Since there is a close link between CSCs, hypoxia and the described signaling pathways, we will also discuss the use of some of these inhibitors to improve the radiosensitization of CSCs to charged particle irradiation and inhibit the tumor hypoxia induced signaling pathways. Next to that, we will also focus on the optimization of treatment delivery techniques to overcome hypoxia-induced radioresistance.

**Table 3:** Preclinical studies investigating possible radiosensitizers in combination with particle RT.

	**Target**	**Small molecule inhibitor**	**Type of irradiation**	**Dose range**	**Time of drug administration**	**Experimental model**	**References**
Hypoxia/HIF/VEGF	VEGF	Bevacizumab	Proton	12, 16 Gy	24 h before irradiation	HTB140 (Melanoma)	(83)
EGFR/PI3K/MAPK pathway	EGFR	Cetuximab	Carbon ion	1, 2, 3, and 4 Gy	1 h before irradiation	SQ20B (Human laryngeal squamous cell carcinoma)	(84)
	mTOR	Temsirolimus	Carbon ion	0.125, 0.5, 1, 2, and 3 Gy	4 h before irradiation	Hep3B, HepG2, PLC, HuH7 (Human liver cancer cells)	(93)
	ERK1/2	PD98059	Pulsed proton beam	4 and 10 Gy	3 h before irradiation	CSC of MCF-7 and MDA-MB-231 (Human breast cancer cells)	(92)
	P38 MAPK	SB203580	Pulsed proton beam	4 and 10 Gy	3 h before irradiation	CSC of MCF-7 and MDA-MB-231 (Human breast cancer cells)	(92)
	EGFR	Gefitinib	Proton beam	3 and 6 Gy	NA	NSCLC H460 and H1299 cells (Human non-small Cell Lung Cancer Cells)	(85)
PARP	PARP1/2	AZD2281 (Olaparib)	Proton	1-6 Gy	2 h before irradiation	A549 (Human lung cancer cells), MIA PaCa-2 (Human pancreas cancer cells)	(96)
		Olabarib B02 (RAD51 inhibitor)	Proton	0.5, 1, 2, and 3 Gy	3–4 h before irradiation, total duration of 24 h	A549 (Human non small-cell lung cancer cells) KP4 and PANC1 (Human pancreatic cancer cells)	(171)
		Olaparib	Carbon ion	1, 3, and 5 Gy	2 h before irradiation	MIA PaCa-2 (Human pancreas cancer cells)	(98)
		PARP-1 knockdown	Carbon ion	1, 2, and 4 Gy	Before irradiation	HeLa (Human cervix carcinoma cells)	(97)
		Talazoparib Olaparib AG14361	Carbon ion	2 Gy	2 h before irradiation, until the end of the experiment	R633, TG1 (Human glioblastoma stem-like cells)	(99)
Hh	GLI1/2	GANT61	Carbon ion	0, 0.25, 0.5, 1, 2, 3, and 4 Gy	72 h before irradiation	PC3 and DAOY (Prostate cancer cells Pediatric medulloblastoma cells)	(102)
	GLI1/2	GANT61	Proton	0.25, 0.5, 2, 4, and 6 Gy	72 h before irradiation	PC3 and DAOY (Prostate cancer cells Pediatric medulloblastoma cells)	(102)
	GLI1/2	GANT61	Carbon ion	0, 0.25, 0.5, 1, 2, 3, and 4 Gy	72 h before irradiation	MCF-7 (human breast cancer cells)	(170)

*HIF, hypoxia-inducible factor; VEGF, vascular endothelial growth factor; EGFR, epidermal growth factor receptor; PI3K, phosphoinositide 3-kinase; MAPK, mitogen-activated protein kinases; PARP, Poly (ADP-ribose) polymerase; CSC, cancer stem cells*.

### Hypoxia

#### Counteracting Hypoxia-Induced Radioresistance by Inhibition of HIF-1 or VEGF

Hypoxia-induced radioresistance can be counteracted by the inhibition of HIF-1, since this protein plays an important role herein. Some HIF-1 inhibitors have been developed and tested in combination with conventional radiotherapy. The results for this combination-therapy have been controversial, with some studies observing no radiosensitizing effect and other studies observing a suppressed tumor recurrence after treatment (172–175). Combining particle therapy and HIF-1 inhibitors has not been investigated so far. However, despite the fact that protons have a similar OER as photons, evidence shows that protons decrease the expression of HIF-1α in comparison to photons (50), hence the use of HIF-1 inhibitors might be redundant in combination with protons. Due to the high RBE and low OER, carbon ions are known to be more effective in damaging hypoxic cells. In addition, evidence of carbon ion irradiated cells already showed decreased expression levels of HIF-1α (53, 54). In this regard, combining HIF-1 inhibitors with carbon ions could have no added therapeutic value, but combination studies are warranted to make a final conclusion. Finally, the use of HIF-1 inhibitors in combination with photon therapy has been controversial, due to the complexity of this pathway. Moreover, most HIF-1 inhibitors target the HIF-1 pathway indirectly and the timing of HIF-1 inhibitor administration (before, during or after radiotherapy) can also affect the outcome. Therefore, tackling of the HIF-1 pathway might be challenging in combination with particle therapy (176).

Several studies already investigated the combination of VEGF inhibitors (e.g., bevacizumab) with conventional radiotherapy, and many of these studies observed a radiosensitizing effect (177, 178). For particle irradiation, only one *in vitro* study has been published combining bevacizumab with proton irradiation (Table 3). In this study bevacizumab was able to increase the radiosensitivity of melanoma cells to proton irradiation compared to photon irradiation in the absence of the drug (83). While conflicting results exist to whether VEGF expression increases or decreases after carbon ion irradiation, no studies investigating the combination of VEGF inhibitors with carbon ion irradiation have been published so far (55–57). However, since carbon ion therapy is used for more radioresistant tumors, the addition of a VEGF inhibitor might be even more beneficial. Specifically, since VEGF is also important in metastasis, its inhibition could have an additional advantage to potentially reduce the migratory potential of the radioresistant tumor cells even more.

#### Dose- and LET-Painting to Overcome Hypoxia

In order to overcome radioresistance due to tumor hypoxia, the concept of dose painting has been introduced in which the radiation dose is increased in hypoxic subvolumes within the tumor. However, this creates several challenges in conventional radiotherapy. Based on the OER of photons, a factor three higher dose is required to eradicate hypoxic cells compared to fully oxygenated cells. This requires very steep dose gradients, which are difficult to achieve with photons. The better dose confirmation of protons and carbon ions and the decrease of OER with LET, make them an attractive solution to alleviate some of the problems posed by hypoxia dose painting with photons (179). In addition to dose painting, proton and carbon ion therapy may also offer the possibility of modulating the LET over the tumor: the so-called LET painting (180, 181). Cancer cell eradication could be maximized if LET could be redistributed according to the spatial hypoxia profile in radioresistant tumors. In others words, it would be beneficial if the high-LET components of the distal part of the proton Bragg peak or carbon ions beam can mainly be co-localized with hypoxic regions. Despite the large amount of preclinical research on both dose- and LET-painting and the improved identification of hypoxic cells in tumors by the help of new imaging and physiological techniques, the real use of hypoxia information in the clinics is still missing (182). Several modeling studies demonstrated the potential higher tumor control probability that can be obtained with dose- and LET-painting with charged particles. Dose-painting resulted in higher tumor control probability compared to LET-painting, particularly for protons, while LET-painting also provided better results for carbon ions compared to conventional carbon ion therapy with no LET- or dose-painting (179). Based on experimental data of OER at intermediate levels of oxygen concentration and LET, an OER model was validated and implemented in an ion treatment planning system TRiP98 (183). This so called “kill-painting” approach would contribute to biologically driven treatment planning, in which the biological effective dose is optimized in the local tumor microenvironment. This model has recently been expanded with the capability of handling different ion beams simultaneously (MIBO version), where 3D target oxygenation data can be used in the treatment planning system (184). Although several simulation studies show an increase in tumor control probability with charged particle therapy for radioresistant hypoxic tumors by applying dose- and/or LET-painting, further validation of these models is needed before they can be implemented in clinical practice. In addition, it is important to take into consideration that LET-painting might increase the normal tissue dose. In order to overcome this problem, an interesting new strategy might be to perform multi-ion LET painting, where low- and high-LET radiation is combined to obtain dose and LET conformity in the tumor (184, 185).

### Inhibition of EGFR Pathway

Cetuximab, an antibody targeting EGFR, is a well-known and widely-used anti-cancer monotherapy in clinical practice. The combination of cetuximab with photon irradiation has been investigated both *in vitro* and *in vivo*, illustrating its ability to sensitize cancer cells to conventional radiation (186–190). This resulted in a phase III clinical trial where head and neck squamous cell carcinomas patients showed an improved clinical outcome when treated with conventional radiotherapy in combination with cetuximab (191). Other inhibitors that target the tyrosine kinase domain of EGFR show also promising results for combined use in conventional radiotherapy (192–194). In the study of Park *et al*. it was found that the EGFR-inhibitor Gefinitib repressed DNA repair after proton irradiation, making them more prone to cell death when compared to photon-treated cells (85). So far, only one study investigated the radiosensitizing effect of an EGFR inhibitor in combination with carbon ion irradiation. In this study, cetuximab was not able to sensitize head and neck CSCs *in vitro* to carbon irradiation compared to carbon ion irradiation alone (84). It should be noted that in this paper, no colonies were observed after the combination of cetuximab and carbon ion radiation, which the authors contribute to the cytoxicity of cetuximab. In addition, the SQ20B cells that were used in this study overexpress EGFR, so these results should be interpreted with caution. Furthermore, the combined use of cetuximab, intensity-modulated radiotherapy (IMRT) and a carbon ion boost has been investigated for its toxicity and efficacy (195, 196). Unfortunately, these phase I/II studies were terminated due to the low number of patients recruited.

Numerous inhibitors of the PI3K pathway, which is one of the downstream targets of EGFR, have been developed and extensively tested in combination with photon irradiation. The PI3K pathway can be targeted at the level of PI3K, AKT and mTOR and even dual inhibitors of the pathway exist, targeting both PI3K and mTOR. The general response that has been observed for the combination of PI3K pathway inhibition with photon irradiation is an enhanced sensitivity of the cancer cells to radiation (197–201). No studies have investigated the combination of PI3K pathway inhibitors with proton irradiation. In contrast, for carbon ion irradiation the mTOR inhibitor Temsirolimus was not able to sensitize hepatic cancer cells (Table 2) (93). The lack of increased sensitization compared to carbon ion irradiation alone can be explained by the previously published evidence that carbon ions decrease the activity of the PI3K pathway and therefore the additional inhibition of this pathway does not result in extra radiosensitization. However, previously discussed studies showed that protons are able to activate EGFR signaling, so it would be interesting to test PI3K inhibitors in combination with proton radiation.

Several MAPK pathway inhibitors have been developed, targeting RAF, RAS, ERK, or MEK. Inhibitors of Ras have been combined with photon radiation and most have proven to be radiosensitizing, both *in vitro* and *in vivo* (128, 202, 203). For particle irradiation, inhibition of ERK, or p38 MAPK was able to sensitize breast CSCs to proton irradiation (92). The sensitizing effect of MAPK pathway inhibitors was expected since activation of EGFR and the MAPK pathway has been observed in response to proton radiation (51, 85, 129). However, besides their dosimetric advantage, the potential added value of protons was not evaluated since the study did not compare the results of the MAPK inhibitors in combination with proton irradiation to conventional photon irradiation. To the best of our knowledge, the combination of MAPK pathway inhibitors with carbon ion irradiation has not been investigated so far. Although current data show that carbon ion irradiation is most likely to decrease the expression of the MAPK pathway, it would be interesting to further investigate the combination of carbon ion irradiation with inhibition of the MAPK pathway especially in tumors with mutations in the MAPK pathway.

### Inhibition of NFκB Pathway

Currently, more than 750 inhibitors of the NFκB pathway have been developed (204). Despite this abundance of NFκB inhibitors, only few have been tested *in vitro* in combination with conventional radiotherapy. The overall outcome of these studies suggests that radiosensitivity can be induced by inhibiting the NFκB pathway (205–209). Unfortunately, the combined use of NFκB inhibitors with particle irradiation has not been explored yet. As evidence has shown, the NFκB pathway can become activated upon irradiation with carbon ions. Therefore, it would be of interest to test NFκB inhibitors in combination with carbon ion radiation. Specifically, since the combination of charged particles with NFκB inhibition would also offer an added dosimetric advantage compared to the combination with photons. So far, the impact of proton irradiation on the NFκB signaling pathway remains unclear. Therefore, more studies are needed to increase our understanding of the effect of proton irradiation on the activation of the NFκB pathway and the potential effect of a combination treatment with NFκB inhibitors.

### Inhibition of PARP

The PARP protein is important in DNA repair and is therefore a promising target for radiosensitization of resistant tumors. Many PARP inhibitors have been developed of which three (olaparib, rucaparib, and niraparib) have been approved by the Food and Drug Administration (210). The combination of PARP inhibitors with photon irradiation has been investigated extensively in preclinical studies and phase I and II clinical trials (211). Results from preclinical studies show that PARP inhibition is an effective target for enhanced sensitivity to photon irradiation, and could even circumvent hypoxia-induced radioresistance. However, the results from clinical trials are conflicting, with some even reporting negative results.

In a study with proton irradiation, the inhibition of PARP resulted in the sensitization of lung cancer and pancreatic cancer cells, due to the disturbed DNA damage response (96). When both PARP signaling and homologous recombination was inhibited, lung cancer and pancreatic cancer cells showed enhanced proton radiation-induced cell killing (171). For carbon ion irradiation, multiple *in vitro* studies have shown that the use of a PARP inhibitor can sensitize cancer cells to carbon ion irradiation (Table 3) (97, 98, 212). Interestingly, some of these studies showed that PARP inhibition has a higher radiosensitizing effect in combination with carbon ion radiation compared to photon irradiation (97, 98, 101). Overall, combining particle radiation with PARP inhibition shows very promising results concerning radiosensitization. In addition, the fact that particle radiation induces more complex DNA damage compared to photons can be an advantage. By additionally inhibiting PARP, repair of more complex DNA damage will be hampered and cells will more likely go into apoptosis instead of cell cycle continuation.

### Inhibition of Hedgehog Pathway

Hedgehog signaling can be inhibited at different stages of the pathway and many Hh pathway inhibitors have been developed over the years. Some preclinical studies performed experiments combining Hh inhibitors with photon irradiation. Hh inhibitors cyclopamine, LDE225 and GANT61 have been reported to sensitize cancer cells *in vitro* and *in vivo* to photon irradiation (115, 165, 213–215). Several small clinical studies investigated the combination of photon irradiation with the Hh inhibitor vismodegib and reported that this combination has radiosensitizing effects in basal cell carcinomas (216–218). In addition, Hh signaling is an important pathway in CSC evolution and may have an impact on the intrinsic radioresistance of these cells. Several issues regarding the precise role of Hh signaling in CSC remain unresolved at this point, but a growing number of studies investigates the clinical use of Hh inhibitors to target CSC (219), which could also have a positive impact on their radioresistance. Despite the lack of prior knowledge on the effect of particle irradiation on the Hh pathway, it would be of interest to investigate this further. Specifically, for Hh driven cancers, such as medulloblastomas or basal cell carcinomas where the combination of Hh inhibition with particle therapy could be of interest for the patient. For example, sonic Hedgehog-driven medulloblastomas are mostly observed during infancy or adult life. The treatment involves craniospinal irradiation (CSI) and chemotherapy to prevent metastasis. However, CSI involves many challenges for conventional radiotherapy due to the close proximity of the organs at risk. Therefore, particle therapy in combination with Hh inhibitors could offer an additional benefit for these patients due to the superior targeting. In a recent study, inhibition of Hh signaling radiosensitized medulloblastoma cells to both proton and carbon irradiation (102).

## Discussion and Open Questions

Despite the many advances made in the field of conventional radiotherapy, radioresistance is still observed and has an unfavorable, adverse or even harmful impact on the outcome of cancer patients. The dosimetric advantage of charged particle therapy has led to a vast increase in the number of patients treated with charged particles for specific clinical indications. However, little is known on their impact on underlying aspects of the tumor microenvironment and molecular mechanisms involved in radioresistance.

Carbon ions offer a clear benefit to overcome hypoxia-induced radioresistance due to their low OER. For protons however, this is less obvious since they have a similar OER as photons. However, some studies have observed a decreased expression of HIF-1α and VEGF after proton irradiation. This could therefore be an advantage in comparison to photons in the treatment of hypoxic tumors. While there are some studies available on the use of HIF-1 inhibitors in conventional radiotherapy with varying results, no one investigated the inhibition of hypoxia related pathways in combination with proton radiation. Due to a lack of these studies, there is no evidence so far on the potential benefit of combining HIF-1 inhibitors and proton therapy for hypoxic tumors and therefore future research is urgently warranted. Besides this, particle therapy offers additional options to overcome hypoxia-induced radioresistance, more specifically by applying dose- and LET-painting. The redistribution of the high-LET region of the distal part of the Bragg peak by dedicated treatment plan optimization allows to maximize LET in specific regions of the target volume. Dose-painting in intensity-modulated proton therapy seems promising and provides similar dose conformity of the dose-painted target compared to IMRT. However, the additional benefit of dose-painting protons is the significant reduction of radiation dose to surrounding normal tissue (220). While both dose- and LET-painting seem to be beneficial in carbon therapy, dose-painting looks more promising than LET-painting for proton therapy. However, more fundamental biophysical research is needed to bring this to clinical practice. In this context, the use of dose- and LET-painting in combination with HIF-1 inhibitors might provide promising perspectives for the treatment of radioresistant hypoxic tumors with charged particles.

Targeting CSCs has gained significant interest in cancer treatment, since it is believed that CSC eradication is essential for a successful treatment. A handful of studies have shown that particle radiation (protons or carbon ions) is more effective against CSCs than photon radiation, due to the increase in ROS production in the case of proton irradiation and the additional beneficial effect of carbon ions on the radioresistant hypoxic niche of CSCs. Since CSCs have distinct intrinsic mechanisms that are responsible for their enhanced radioresistance, more fundamental research is needed to understand the full extent of particle radiation on the outcome of DNA repair and survival in CSCs. Future research should preferably focus on the combined use of charged particle radiation with inhibitors that target specific pathways involved in CSCs radioresistance.

Several molecular pathways in cancer cells have been implicated in resistance to photon radiation. Whether these pathways also concur resistance to particle radiation is currently unclear. In addition, many molecular inhibitors have been tested in combination with conventional radiotherapy, while only very few have been tested in combination with protons or carbon ions. Since particle therapy is on the rise, this calls for the further exploration of these combination therapies in a preclinical setting. Previously, particle radiation facilities provided limited access for biological experiments, which limited the time to perform such experiments. However, international consortia on particle therapy research, such as the European Network for Light ion Hadron Therapy (ENLIGHT—https://enlight.web.cern.ch/) and the well-established Particle Therapy Co-Operative Group (PTCOG—https://www.ptcog.ch), are growing and recognize the potential of radiobiological experimental work. Therefore, the European Particle Therapy Network is investing a lot of efforts to form a network of research and therapy facilities in order to coordinate and standardize radiobiological experiments (221). For carbon ions specifically, limited data on combination therapies are available. This is mainly due to the high RBE of carbon ions by which the additional benefit of molecular inhibitors might be difficult to demonstrate. Furthermore, the use of carbon ions worldwide is limited, which could also explain why fewer studies have been published regarding combination treatment with carbon ions. However, it is important to consider that although several molecular inhibitors have been tested in combination with photon radiation, many did not result in an increased radiosensitizing effect. This could be due to improper preselection of cell lines in which the chosen pathway to inhibit is not active. Therefore, a careful selection of the experimental set-up is crucial in future combination experiments with pathway inhibitors and particle radiation. In addition, a proper comparison with conventional photon irradiation is advisable, in order to have a clear idea of the additional biological advantage proton and/or carbon ion therapy might bring next to the dosimetric advantages.

From literature it is clear that there is a huge variety in physical parameters used in the charged particle radiation experiments, reflected by the range of beam energies and LET values in Table 2, for this type of fundamental radiobiological research. In addition, the experimental models range from *in vitro* normal cells to cancer cells and *in vivo* models. While it is anticipated that an experimental condition should be tested on more than one cell line *in vitro*, it is important to select which is relevant to clinical practice. This wide variety in experimental models and designs, together with the limited number of studies could be an explanation for some of the contradictory results that are observed. Therefore, future experiments should first of all use clinically relevant beam qualities. For example, in proton therapy, the energy is directly related to the depth in tissue and the therapeutic range starts at ~70 MeV, for superficial tumors, up to 250 MeV for deeper-seeded tumors in the human body. In addition to the different energies and LET values that are used, also the drug concentration as well as the time of drug administration can have an influence on the final result. Cell lines derived from different tissue types have variable half maximal inhibitory concentration (IC_50_) values which can also influence the drug concentration that is eventually chosen for further experiments. As previously mentioned, it is important to select cell lines for *in vitro* experiments that represent a clinical indication for particle therapy when one aims to study the signaling pathways. In addition, more attention should be paid to CSCs in future preclinical studies. However, the tumor microenvironment is in reality much more complex than the *in vitro* 2D cell cultures and growing evidence supports that future research efforts should focus on 3D *in vitro* models and animal models. These animal studies are particularly important to design combination therapies with molecular targeting and fractionated particle irradiation. For instance, the inhibition of HIF-1 at an unsuitable timing can suppress rather than enhance the effect of radiation therapy because its anti-angiogenic effect increases the radioresistant hypoxic fraction. While the first *in vitro* results illustrate different effects for charged particles compared to photons, it remains important to determine the treatment regimen and critical timing of HIF-1 inhibitor administration to enhance rather than inhibit the therapeutic effect of radiation. Despite the fact that *in vitro* models can provide valuable insights and a first indication of the effectiveness of the combined therapy, pre-clinical animal studies with charged particles are warranted to get a better understanding of how the combination therapy should be designed. Preclinical animal studies with charged particles present an important gap in current scientific literature and such studies are urgently needed to broaden the clinical scope of charge particle therapy in combination with molecular targeting.

In conclusion, more research needs to be performed regarding the potential superior role of particle therapy in treating radioresistant cancers. An in-depth evaluation of the impact of carbon ion and proton irradiation on hypoxia, the potential to eradicate CSCs and the potential targetable pathways in combination with particle radiation is warranted. Since the current clinical-base supports the use of particle therapy in a limited range of clinical indications, this research might broaden the clinical scope of particle therapy, especially in the context of radioresistant tumors. In addition, novel molecular strategies were designed to interfere with the molecular pathways involved in radioresistance. Moreover, several clinical trials have been undertaken to evaluate their effect in conventional photon-based radiotherapy, but only a limited number of studies evaluated the use of these inhibitors in particle therapy so far. Furthermore, this review highlights the wide variety in the experimental conditions of *in vitro* studies that have been performed up to now and calls for clinically relevant experimental designs in order to get a more uniform body of results. Lastly, *in vivo* experiments are necessary to confirm the most promising *in vitro* results in relation to new potential advantages of particle therapy to overcome radioresistance.

## Author Contributions

KK, CV, and MM wrote the draft manuscript. CV, BB, and SB revised the manuscript. MM, BB, CV, and SB acquired funding. All authors read and approved the manuscript.

### Conflict of Interest

The authors declare that the research was conducted in the absence of any commercial or financial relationships that could be construed as a potential conflict of interest.
